# Pharmacotherapy of *Demodex*-Associated Blepharitis: Current Trends and Future Perspectives

**DOI:** 10.3390/pharmacy13050148

**Published:** 2025-10-15

**Authors:** Aleksandra Czępińska-Myszura, Małgorzata Maria Kozioł, Beata Rymgayłło-Jankowska

**Affiliations:** 1 Clinic Department of Diagnostics and Microsurgery of Glaucoma, Medical University of Lublin, Chmielna 1, 20-400 Lublin, Poland; ola.czepinska@wp.pl (A.C.-M.); beata.rymgayllo-jankowska@umlub.edu.pl (B.R.-J.); 2 Students Scientific Association, Department of Medical Microbiology, Medical University of Lublin, Chodzki 1, 20-093 Lublin, Poland; 3 Department of Medical Microbiology, Medical University of Lublin, Chodzki 1, 20-093 Lublin, Poland

**Keywords:** DAB, ocular infections, eye parasitosis, treatment, ophthalmologist, pharmacists, patient’s education

## Abstract

*Demodex*-associated blepharitis (DAB) is a common condition in our society. Patients report not only uncomfortable and bothersome symptoms but also decreased self-esteem and confidence. Because of its nonspecific signs, pharmacists are often the first healthcare professionals patients consult. What is most concerning is that DAB can cause serious complications within the eye, such as dry eye syndrome, corneal scarring, or recurrent styes and chalazia. Therefore, we aimed to compile both standard and innovative therapies and compare their effectiveness and safety. As shown, standard methods remain the recommended approach. Alongside antiparasitic agents such as metronidazole or ivermectin, education and improved eyelid hygiene are crucial. However, in recent years, promising new treatments for *Demodex* blepharitis have emerged, such as Lotilaner Ophthalmic Solution 0.25%, which has shown positive results in clinical trials. Mechanical techniques, including Intense Pulsed Light (IPL) therapy and eyelid peeling procedures such as Blepharoexfoliation, have also demonstrated promise. Due to the notable effects of tea tree oil, studies have explored the lethal effects of other essential oils, such as sage, peppermint, and bergamot oils. These are just a few of the interesting examples discussed in this paper.

## 1. Introduction

### 1.1. The Parasite

**Figure 1:**
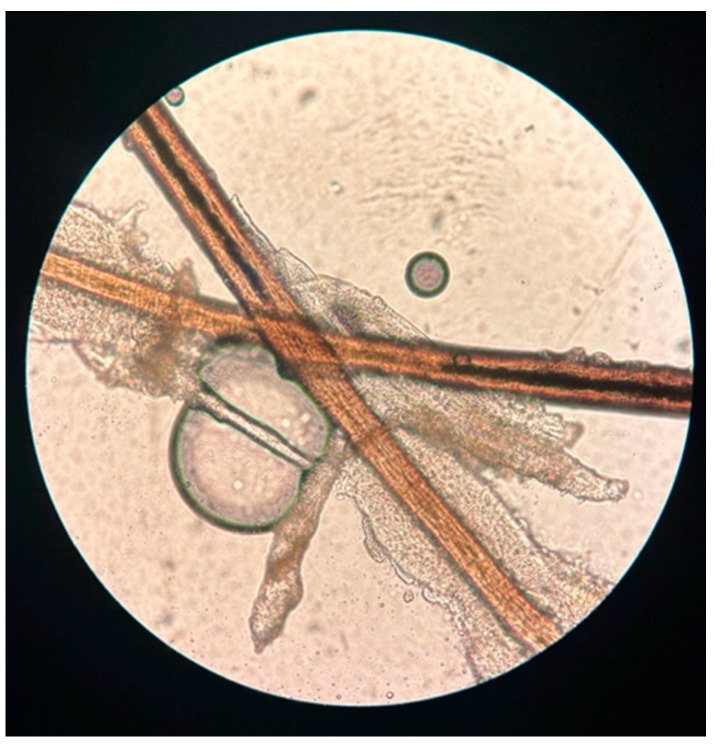
The microscope photograph shows a few mites from the *Demodex* spp. group on two epilated eyelashes (the photo is property of the Clinic Department of Diagnostics and Microsurgery of Glaucoma, Medical University of Lublin).

*Demodex* spp. is a mite from the arachnid group that often colonizes human skin (Figure 1). There are two main species: *Demodex brevis* and *Demodex folliculorum*. On the face, it prefers areas around the nose, cheeks, forehead, and eyelids. In men, it can also inhabit the hairy skin of the beard and chest. Residing in sebaceous glands, hair follicles, or Meibomian glands on the eyelids, it feeds on sebum and epidermal cells [1,2]. During infestation, it excretes waste and secretes substances that irritate the delicate skin, plug hair follicle ducts, and intensify hyperkeratinization. Then, hypersensitivity is activated by pro-inflammatory cytokines, like IL-1b and IL-17, and enzymes such as matrix metalloproteinase-9 (MMP-9) [3]. Moreover, as recently shown in a study by Pyzia et al., *D. folliculorum* can act as a vector for bacteria such as *Staphylococcus aureus, Acinetobacter baumannii, Streptococcus pneumoniae, Klebsiella oxytoca, Corynebacterium* spp., and *Bacillus* spp.. Alongside patients’ comorbidities, they can lead to serious ocular superinfections [4].

### 1.2. Epidemiology and Risk Factors

**Figure 2:**
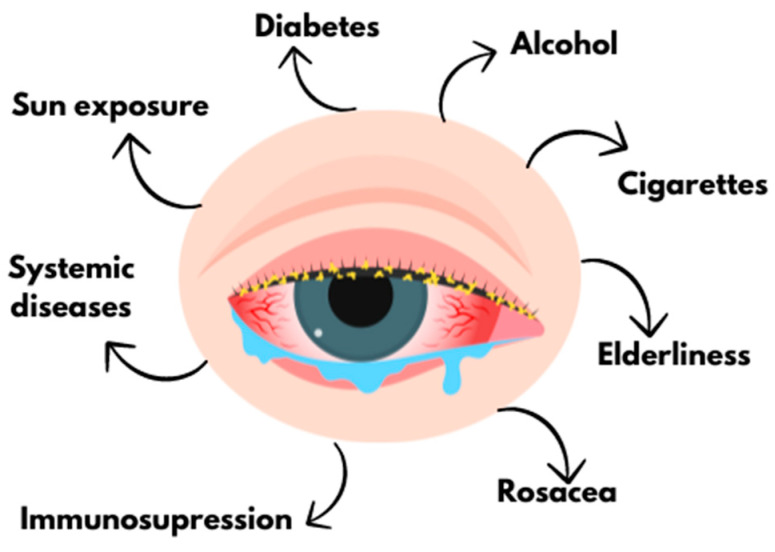
Risk factors of ocular demodicosis (created with Canva.com (https://www.canva.com, accessed on 31 May 2025) based on Refs. [1,6]).

Referring to the introduction, one of the most common and troublesome ophthalmic problems is demodectic blepharitis. It is estimated that this condition affects more than 80% of people over the age of 60 and up to 100% of the population over the age of 70 [5]. Indeed, older age is one of the main risk factors, but not the only one; other triggers include, for example, diabetes, rosacea, alcohol abuse, smoking, sun exposure, and the use of systemic or topical corticosteroids [1]. As the literature shows, demodicosis also easily develops in people with reduced immunity due to the use of immunosuppressive drugs or systemic diseases, such as leukemia, lymphoma, or Acquired Immunodeficiency Syndrome (AIDS) [6] (Figure 2).

### 1.3. Clinical Features

**Figure 3:**
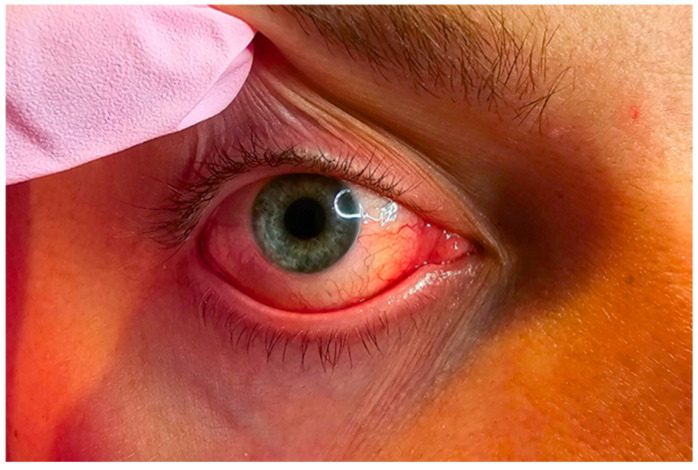
A photograph of a patient’s eye with chronic ocular demodicosis, characterized by redness of the conjunctiva, itching, tearing, excessive eyelash loss, and morning eyelash sticking (the photo is property of the Department of Medical Microbiology, Medical University of Lublin).

*Demodex*-associated blepharitis (DAB) causes a wide range of symptoms, such as redness of the conjunctiva and eyelid margins, dry eye syndrome, burning, itching (especially at night or in the morning), pain, tearing, excessive eyelash loss, morning eyelash sticking, and the condition’s pathognomonic sign—collarettes on the eyelashes, like cylindrical dandruff [2,7] (Figure 3). These symptoms are nonspecific for most people; so, they often ignore the problem and do not consult an ophthalmologist immediately. Instead, they turn to pharmacists, seeking conservative home remedies.

### 1.4. Psychosocial Impact

Moreover, patients often report a decrease in self-esteem and quality of life due to these symptoms. Data indicate that blepharitis negatively affected 80% of patients. The symptoms hindered maintaining eye hygiene (30%), wearing contact lenses or makeup (34%), and driving at night (47%) [8]. Additionally, a multicenter study involving 524 participants ranked the three most bothersome symptoms as itchy eyes, dry eyes, and foreign body sensation. Furthermore, 32.3% of patients visited a doctor for blepharitis at least twice, including 19.6% who saw a specialist at least four times [9]. These findings suggest that DAB is often misdiagnosed, leading to delayed treatment and worsening of the condition.

### 1.5. Complications

It was found that ocular demodicosis is not just a cosmetic issue or a source of uncomfortable symptoms. It can cause serious problems in the eye’s protective structures and its front segment, such as tear film disorders or recurrent chalazia and styes, which sometimes require surgery. Concerning the cornea, infestation by these mites and ongoing irritation can lead to keratopathy, pathological vascularization, clouding, or nodular scarring [10]. Interestingly, preliminary observations by Kurtul et al. even indicated a link between keratoconus and demodicosis. This may be due to constant rubbing caused by chronic inflammation and the symptoms mentioned above [11]. Naturally, more research is needed to confirm this.

### 1.6. Treatment

Current management strategies mainly rely on symptomatic treatment, lid hygiene, and the use of tea tree oil (TTO) or essential oil derivatives, but they face issues like poor long-term adherence and inconsistent efficacy and tolerability [12]. Therefore, developing new techniques for eradicating and treating *Demodex* blepharitis is essential for improving patients’ quality of life. These methods should be effective, free of side effects, easily accessible, and affordable. Is this feasible? New approaches, including exploring alternative pharmacological agents and innovative formulations, have been reported, although clinical evidence is still emerging.

### 1.7. The Aim

Consequently, this paper aimed to gather available and innovative methods for treating DAB and to compare their effectiveness. It also emphasized the importance of patient education regarding at-home eyelid hygiene procedures and highlighted the crucial role of pharmacists, who are often the first healthcare professionals patients consult.

The analysis was based on papers from the PubMed, Web of Science, and Scopus databases. The following inclusion criteria were used in this review: publication date, reliability of the information, and compliance with the topic.

## 2. Standard Treatment

### 2.1. Eyelid Hygiene and the Role of Pharmacists

The primary and essential method for managing *Demodex* blepharitis is maintaining proper eye hygiene, which includes applying warm compresses to the eyelids, cleansing the eyelashes with specially designed disposable wipes, or at least using gentle baby shampoo. Regular eyelid massage is also recommended to ensure adequate drainage of the Meibomian glands [12,13]. Currently, numerous anti-*Demodex* products are available in pharmacies, including wipes, special liquids or gels, and compresses with antibacterial coatings, which can be easily warmed in the microwave [14]. Therefore, pharmacists should be knowledgeable about their proper use to instruct patients appropriately. This is especially important since they are often the first healthcare professionals people approach. Pharmacists should also be able to recognize when to refer a patient to a specialist due to the ineffectiveness of conservative, home-based management. However, in most cases, eye hygiene alone is not enough to eliminate the mites. Pharmacological agents, natural substances, or specialized procedures are necessary [13].

### 2.2. Antiparasitic Agents

Commonly, antiparasitic medications and antibiotics, such as metronidazole (MTZ) and ivermectin, are used systemically and/or topically. Ivermectin acts by binding selectively to glutamate-gated or γ-aminobutyric acid–gated chloride channels in the peripheral synapses of neurons. The primary result is the inhibition of the parasite’s nerve and muscle cells, leading to paralysis and ultimately death [3]. Then, the MTZ acts by reducing and transitioning to nitro radicals. These metabolites, such as N-(2-hydroxyethyl) oxamic acid and acetamide, can react with DNA and form adducts with guanosine [15], causing damage to *Demodex*’s genome and leading to death.

In the study by Choi et al., the efficacy of topical ivermectin 1%, used once a week for 15 min over approximately 15 weeks, in conjunction with eye hygiene, showed a significant reduction in redness, eyelid swelling, and telangiectasia [16]. However, in treatment-resistant cases, the most effective pharmacotherapy is a combination of ivermectin and MTZ used simultaneously. Salem et al. reported that patients receiving both agents for four weeks achieved faster and higher remission rates compared to those treated with ivermectin alone [17].

However, it should be noted that oral therapy with ivermectin and/or metronidazole is associated with potential adverse effects, including nausea, abdominal pain, and diarrhea. In rare cases, neurotoxicity, optic neuropathy, or encephalopathy can even happen [15]. Moreover, relapses following antiparasitic treatment are relatively common [18]. Other antiparasitic agents, such as permethrin, crotamiton, benzyl benzoate, and tetracyclines, have also been used. But their efficacy remains inconsistent, and they pose a higher risk of irritation and side effects [19,20].

### 2.3. Tea Tree Oil

Therefore, in the development of pharmacotherapeutic methods, focus has been given to tea tree oil, which contains terpinen-4-ol (T4O), as a natural alternative. The full mechanism of TTO against *Demodex* mites remains unclear. It causes the parasite to migrate out of the hair follicles. More specifically, T4O has acetylcholinesterase-inhibiting effects that produce the acaricidal impact [13,21].

It can be applied in the form of wipes, shampoos, eyelid gels, or through direct massage in concentrations ranging from 5% to 50%. It should be noted that higher concentrations are used less frequently (e.g., once a week) due to an increased risk of ocular irritation [21].

**Figure 4:**
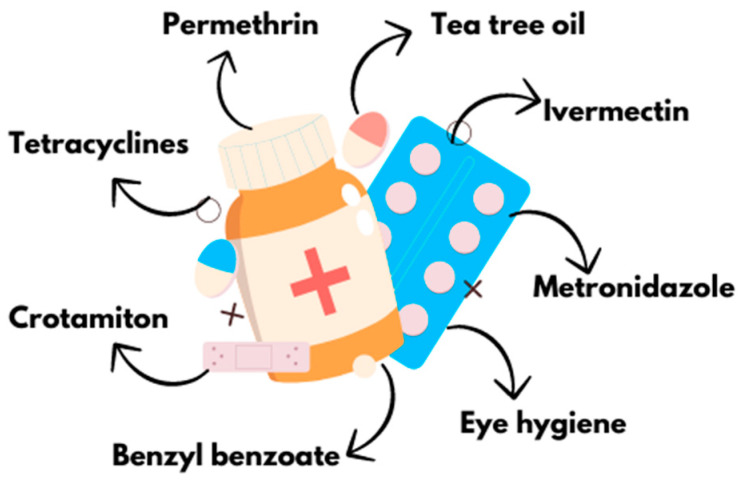
Classic therapies against ocular demodicosis (created with Canva.com (https://www.canva.com, accessed on 31 May 2025) based on Refs. [12,13,14,15,16,17,18,19,20,21,22]).

In a meta-analysis by Savla et al., a significant variation in the effectiveness of this therapy was observed. Although no major side effects were reported, complete eradication was not consistently achieved [21]. Recently, products containing standardized concentrations of T4O (e.g., 2.5% T4O combined with 0.2% hyaluronic acid) have been shown to be well tolerated by patients and to help reduce symptoms or discomfort associated with blepharitis [22] (Figure 4). Therefore, further research and careful monitoring are necessary.

### 2.4. Historical Note

Historically, the literature indicates that in the past, sulfur ointment or yellow mercury ointment were used, but their effectiveness was questionable, and these methods have been abandoned in most countries [23]. Nowadays, they are rarely applied due to the systemic toxicity of mercury compounds and the inability to use them safely for prolonged periods [24].

## 3. Novel Therapies

**Figure 5:**
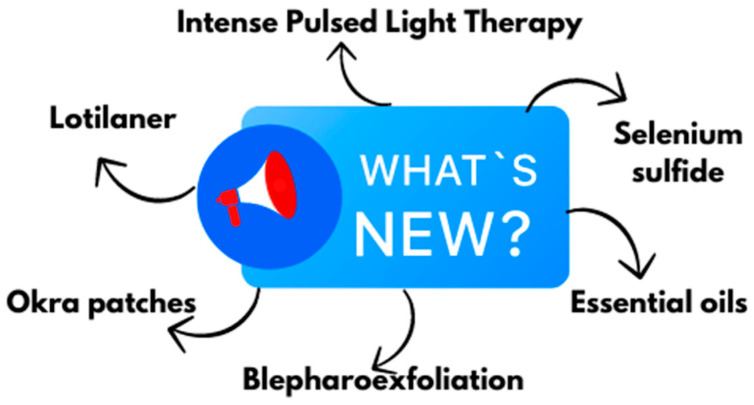
Novel therapies against ocular demodicosis (created with Canva.com (https://www.canva.com, accessed on 31 May 2025) based on Refs. [25,26,27,28,29,30,31,32,33,34,35,36,37,38,39,40,41,42,43]).

*Demodex* blepharitis has gained significant attention in recent years. Researchers from various continents are competing to develop the most effective eradication methods. The market has seen the emergence of artificial chemical substances, mechanical techniques, and natural essential oils. Some of these, such as Lotilaner, have been extensively tested in the United States; others, like blepharoexfoliation and Intense Pulsed Light Therapy, are used sporadically by some centers. There are also interesting alternative substances, such as peppermint oil, sage oil, bergamot oil, okra extract, Povidone-Iodine (PVP-I), or selenium sulfide (Figure 5).

### 3.1. Lotilaner Ophthalmic Solution 0.25%

Lotilaner, an isoxazoline compound initially developed as an oral systemic ectoparasiticide for dogs and cats, acts by inhibiting γ-aminobutyric acid (GABA) receptors and L-glutamate-activated chloride channels in *Demodex* cells, leading to spastic paralysis and death [25,26,27]. Recently, it has been used as the Lotilaner Ophthalmic Solution (0.25%), formerly known as TP-03 (XDEMVY, Tarsus Pharmaceuticals Inc., Irvine, CA, USA). It has been approved by the United States Food and Drug Administration (US FDA) as a treatment for DAB following the completion of randomized, double-blind clinical trials [12,28].

In phase 3 of the study (Saturn-2), involving 412 participants, Gaddie et al. reported that twice-daily application for six weeks led to collarette cure in 56% of patients, mite eradication in 51.8%, and erythema cure in 31.1%. Additionally, 90.7% of participants reported excellent tolerability with minimal adverse effects [29]. Similarly, Talha et al. confirmed the high efficacy and safety profile of Lotilaner Ophthalmic Solution in a meta-analysis, with only mild, transient side effects, such as burning, discomfort, or slight visual blurring [30].

Overall, lotilaner represents a promising and safe therapeutic option for DAB. However, XDEMVY remains difficult to access and expensive in Europe. According to the official website of Tarsus Pharmaceuticals Inc., XDEMVY, a preservative-free formulation, is expected to receive European regulatory approval by 2027 [31].

### 3.2. Intense Pulsed Light Therapy

Intense Pulsed Light (IPL) Therapy, widely used in dermatology and cosmetology since 1992, delivers a broad-spectrum light beam that penetrates the skin and produces thermal energy. Then, chromophores, such as hemoglobin, water, and melanin, absorb the photons and are subsequently heated, leading to selective photothermolysis [32].

Beyond its established indications, including photorejuvenation, scar treatment, pigmentation reduction, and the treatment of chronic inflammatory skin conditions, recently, it has been explored as an option for DAB, mainly since IPL has been successfully used to treat Meibomian Gland Dysfunction (MGD) and dry eye disease (DED) [33]. In vitro observations by Fishman et al. demonstrated that mite exposure to IPL raised their temperature to approximately 49 °C, resulting in immobility and death, confirming the direct acaricidal effect of the therapy [34]. Huo et al. reported that after four sessions of IPL, there was a significant improvement in the ocular surface disease index (OSDI), the tear film’s lipid layer, tear break-up time (TBUT), and the clarity of meibomian gland secretions, accompanied by a marked reduction in mite count and eyelid margin abnormalities [35]. Furthermore, Zhang et al. found that IPL laser achieved faster and more pronounced improvements compared with 5% tea tree oil after one month of treatment [36]. These findings support the idea that this therapy could be used for *Demodex* infestation and MGD.

### 3.3. Blepharoexfoliation

The more advanced method for eradicating *Demodex* is blepharoexfoliation, which mechanically exfoliates debris, mites, and collarettes along the eyelashes, using a rotating head with a micro-sponge. This process also disrupts the bacterial biofilm, which protects the parasites during infestation [37]. Mohammad-Rabei et al. tested the effectiveness of blepharoexfoliation using the BlephEx device, combined with twice-daily peeling with tea tree oil and topical application of erythromycin, for 8 weeks. The study group achieved faster and better results in comparison to the group without BlephEx, as evidenced by decreased OSDI parameters and mite counts [38]. However, current evidence remains limited, and additional controlled clinical studies are required to confirm the long-term efficacy of this approach [37].

To facilitate a clearer understanding of the aforementioned therapeutic approaches, their mechanisms of action, clinical efficacy, adverse effects, and accessibility are summarized and compared in Table 1.

### 3.4. Essential Oils as Natural Alternatives

Since TTO has been recognized for its effectiveness against *Demodex* mites, efforts have been made to investigate other essential oils containing terpenes, which are lethal to mites. Sędzikowska et al. reported that sage oil killed the mites within 7 min, similar to TTO, while peppermint oil took a slightly longer time, killing the mites in 11 min [39]. Additionally, Chudzicka-Strugała et al. mentioned the potential use of castor oil, bergamot oil, and *Nigella sativa* L. seed oil in a synergistic manner [40]. This suggests that the highest efficacy may be achieved by combining these agents into a single formulation. It offers a natural therapy option for patients suffering from *Demodex* blepharitis.

### 3.5. Other Emerging Therapies

In the literature, there are also isolated cases of using Dilute Povidone-Iodine (PVP-I) at 0.25% in dimethyl sulfoxide (DMSO) as a topical gel [41], as well as selenium sulfide combined with carboxymethylcellulose (CMC) or petroleum jelly. In vitro studies by Heczko et al. demonstrated that a 4% concentration of selenium sulfide killed *D. folliculorum* similarly to 50–100% TTO [42]. Furthermore, Liu et al. compared the anti-*Demodex* effects of okra extract and TTO. The results showed that mites exposed to okra-derived substances survived significantly shorter. After three months of treatment, the number of mites and the OSDI index were markedly reduced. However, the overall eradication rate was slightly lower with okra (40.74% vs. 48%), but it caused fewer irritations [43]. These findings suggest that okra-based eyelid patches could be an alternative for patients with sensitive skin. Nevertheless, it is essential to note that while these methods provide alternatives, further in vivo research on a larger scale is necessary.

## 4. Conclusions

When comparing the old and new methods of combating DAB, based on the studies or articles we have cited, it can be stated that only the Lotilaner Ophthalmic Solution 0.25% can compete with standard therapies. Among the innovative options, it is the only one that has been clinically tested on a larger scale and has demonstrated high efficacy in studies, with only mild side effects in a few cases. Currently available options are limited to some countries, and we must wait for their dissemination. IPL therapy and blepharoexfoliation have only been tested on a small group of patients, and some alternative chemical substances have only been tested in vitro. Therefore, further research is necessary to determine which method is the best. They should focus on well-designed, multicenter clinical trials and long-term follow-up studies to validate the safety and sustained effectiveness of emerging therapies, as well as to define standardized treatment guidelines. On the other hand, the popularity of standard treatments depends on the medical center. If demodicosis is not severely advanced, ophthalmologists usually initiate therapy by recommending TTO-based wipes and eye hygiene, and consider pharmacological options only in later stages of treatment.

As was mentioned several times, it is not only ophthalmologists that play a crucial role. Due to nonspecific symptoms, pharmacists are typically the first healthcare professionals patients approach. Therefore, they should stay up to date with the latest anti-DAB products and their proper use to educate people effectively. A good initiative would be to display posters in pharmacies about adequate eyelid hygiene, as well as to distribute new, proven substances for eradicating *Demodex* spp. as soon as they become available on the market.

## 5. Future Directions

*Demodex*-associated blepharitis is a common and serious issue that needs more attention. It not only worsens cosmetic concerns but also leads to dangerous side effects. Therefore, patients should not have to deal with it on their own, and better treatment options must be explored. However, the sensitivity of the eye area to chemicals and mechanical treatments presents specific challenges. According to medical ethics, the benefits of a therapy should outweigh its side effects. So, we face a significant question: “How to effectively and permanently help patients suffering from ocular demodicosis, improve their quality of life, and protect them from the negative effects of infestation, while ensuring comfortable and painless treatment?” Fortunately, in recent years, alongside established methods, new therapies have emerged, some with promising results. They represent a hopeful future in the treatment of DAB. Still, we should continue to develop and test these approaches rather than stopping with current studies. Perhaps one day, we will find a perfect solution.

## Figures and Tables

**Table 1:** Comparison of therapeutic options for *Demodex* blepharitis, including their mechanisms of action, efficacy, reported adverse effects, and current availability.

Treatment Option	Mechanism	Efficacy	Adverse Effects	Availability
**Ivermectine** (topical/oral)	Paralysis of the parasite [3]	Most effective when combined [16,17]	Topical: mild irritation, allergic dermatitis, redness Systemic: more severe interactions and hypersensitivity	Widely available (topical gels/creams; oral tablets)
**Metronidazole** (topical/oral)	Demage of the parasite’s DNA by nitro radicals [15]		Topical: irritation, allergic reaction Systemic: Gastrointestinal upset, very rare: neurotoxicity	Widely available (topical gels/creams; oral tablets)
**Tea Tree Oil/** **Terpinen-4-ol**	Acetylcholinesterase-inhibiting effects [13,21]	Variable efficacy. There is uncertainty related to the effectiveness [21]	Ocular irritation, allergic reaction *(the posibility increases with higher concentration)* [13,21]	OTC wipes/gels/shampoos widely available; various concentrations from 5% to 50% [21]
**Lotilaner Ophthalmic Solution 0.25%** (XDEMVY)	Spastic paralysis of the parasite [25,26,27]	**The highest proven efficacy. The first and only FDA-approved treatment against *Demodex* spp.** [12,28,29,30]	Infrequent: mild burning, discomfort, slight visual blurring; sporadically: chalazion [29,30]	**USA** *(In Europe it is expected to be available in 2027)* [31]
**Blepharoexfoliation** (BlephEx)	Mechanical removal of mites and collarettes [37]	*Unclear *Further multicenter clinical trials are needed*	*Limited data Generally mild discomfort, burning, itching [37]	Offered only by selected eye centers
**Intense Pulsed Light (IPL)** therapy	Photothermolysis [32,33,34]	*Unclear *Further multicenter clinical trials are needed*	*Limited data mild erythema, warmth, discomfort, pigmentation disorders [32,33,34,35,36]	Offered only by selected eye centers

Bold text indicates the treatment with the highest proven efficacy. Italic text denotes author comments or notes where further clarification or data are needed. “*” marks statements requiring additional explanation or where evidence is limited.

## Data Availability

All data are contained within this article.

## Abbreviations

The following abbreviations are used in this manuscript:

DAB	Demodex-associated blepharitis
AIDS	Acquired Immunodeficiency Syndrome
MTZ	metronidazole
TTO	tea tree oil
T4O	terpinen-4-ol
FDA	U.S. Food and Drug Administration
IPL	Intense Pulsed Light
OSDI	ocular surface disease index
TBUT	tear break-up time
PVP-I	Povidone-Iodine
DMSO	dimethylsulfoxide
CMC	carboxymethylcellulose
